# Hydrogel-Based Delivery of Ion Channel Modulators for Cancer Therapy: Current Advances and Future Perspectives

**DOI:** 10.3390/gels12070567

**Published:** 2026-06-26

**Authors:** Gizem Ozkurnaz Civir, Ilknur Atli, Ozgur Ozay

**Affiliations:** 1 Department of Plant and Animal Production, Lapseki Vocation School, Çanakkale Onsekiz Mart University, 17800 Çanakkale, Türkiye; 2 Laboratory of Biomaterials Research, Department of Bioengineering, School of Graduate Studies, Çanakkale Onsekiz Mart University, 17100 Çanakkale, Türkiye; ilknratli@gmail.com; 3 Laboratory of Biomaterials Research, Department of Bioengineering, Faculty of Engineering, Çanakkale Onsekiz Mart University, 17100 Çanakkale, Türkiye; ozgurozay@comu.edu.tr

**Keywords:** hydrogel, ion channel modulators, drug delivery, cancer

## Abstract

Cancer remains one of the deadliest health problems worldwide. Traditional and multifaceted treatment approaches often fail to achieve expected outcomes. Consequently, it is essential to develop innovative and effective treatment strategies. Ion channels play a critical role in regulating fundamental cellular events, such as cell proliferation, differentiation, and programmed cell death. In this context, the current literature increasingly demonstrates the potential of ion channel modulators to suppress tumor growth. Approaches targeting ion channels have gained increasing importance in cancer research in recent years. Drug delivery systems, particularly hydrogels, play crucial roles in enhancing the therapeutic efficacy of these agents. This review addresses the current developments in the antitumor effects of ion channel modulators within the context of hydrogel-based delivery strategies. Considering the pharmacokinetic advantages, controlled release properties, and targeted delivery capacity of these systems, their biocompatibility, stability, and limitations in clinical applications are also evaluated. Thus, innovative perspectives are presented to address the shortcomings in current cancer treatment approaches.

## 1. Introduction

Cancer is a major disease threatening public health due to its high mortality rate. Traditional chemotherapeutic strategies are limited by systemic toxicity, drug resistance, and low specificity. The asymmetric distribution of ions across the cell membrane contributes to the formation of ionic balance, which is necessary for the maintenance of cellular functions. Ion channels are selective transmembrane proteins that regulate the passage of ions across the cell membrane. These channels not only regulate the flow of ions across membranes but also play a critical role in coordinating numerous vital biological processes such as maintaining cellular homeostasis, signal transduction in cancer cells, and regulation of the cell cycle [1].

Ion channels play an important role in regulating fundamental cellular processes, such as cell proliferation, migration, and apoptosis. An increasing number of studies have revealed that ion channel expression and function are frequently impaired in cancer cells. These changes can negatively affect cellular homeostasis, trigger malignant transformation, and consequently contribute to cancer-related processes, such as uncontrolled cell proliferation and invasion. In addition, changes in the tumor microenvironment, such as hypoxia and pH, affect ion channel expression [2].

Evidence regarding the potential of ion channel modulators in cancer treatment has been steadily increasing. Many studies have reported abnormal ion channel expression in different cancer types [3]. The role of ion channels in proliferation, apoptosis, and metastasis suggests that inhibitors or activators of these proteins could be considered as candidate therapeutic agents. Therefore, the delivery of ion channel modulators via drug delivery systems is important for developing more controlled and targeted strategies.

Drug delivery platforms used in cancer treatment are important for therapeutic efficacy and targeted application. These platforms support the selective deposition of therapeutic agents into target tissues while reducing exposure to healthy tissues [4]. Hydrogels offer advantages in terms of controlled drug release owing to their high fluid retention capacity, biocompatibility, soft tissue-like mechanical properties, and sensitivity to environmental stimuli [5]. Recent studies have shown that ion channel modulators can be loaded onto hydrogel-based systems to increase controlled and local drug deposition and develop anticancer effects.

In conclusion, the combination of ion channel modulators and hydrogel-based drug delivery systems is considered an innovative and promising area of research that can contribute to the development of more effective and targeted therapeutic strategies for cancer treatment. Furthermore, the use of ion channel inhibitors or activators in combination with other therapeutic agents may enhance therapeutic efficacy or provide a potentiating effect to overcome drug resistance.

This review discusses the therapeutic potential of ion channel modulators and current approaches to their delivery via hydrogel-based delivery systems. Specifically, Na^+^, K^+^, Ca^2+^, and Cl^−^ channel modulators and their efficacy in cancer progression are evaluated. Furthermore, the effectiveness of hydrogel delivery systems for ion channel modulators is examined, considering current studies, focusing on their contribution to reducing systemic side effects and improving bioavailability. Because this study focuses on hydrogel-based delivery of ion channel modulators, not all modulators in the literature could be evaluated.

## 2. The Role of Ion Channels in Cancer Biology

Ion transport across the cell membrane is an important mechanism for maintaining cellular function and activity [6]. Changes in membrane potential throughout the cell cycle lead to fluctuations in membrane potential, which regulate cell volume and form an important component of proliferative signaling [7].

### 2.1. Ion Homeostasis and Membrane Potential

Ion channels are membrane proteins that regulate the passage of ions across the plasma membrane or intracellular organelles and play critical roles in almost all cellular processes. Transmembrane potential refers to the electrical potential difference that occurs between the two sides of the cell membrane. This potential arises from the selective permeability of the cell membrane and the presence of ion gradients [8].

The asymmetric distribution of ions across the cell membrane leads to the formation of a resting membrane potential (Vm), in which the intracellular space is negatively charged [1]. Changes in the resting membrane potential serve as a critical regulatory mechanism for cell cycle progression. It has been reported that the potential exhibits dynamic fluctuations in breast cancer cells. Furthermore, it has been reported that the membrane potential is more depolarized in tumor cells. This suggests that the relationship between electrophysiological properties and proliferative processes is altered in tumor cells, highlighting ion channels as potential therapeutic targets.

Transmembrane potential and ion balance can contribute to cancer development by influencing processes such as cellular homeostasis, apoptosis, and proliferation [9,10]. Changes in membrane potential have been shown to play a role in regulating processes such as cancer cell proliferation [11,12]. In particular, K^+^ ions play a role in determining the resting membrane potential, whereas Ca^2+^ ions act as critical second messengers in intracellular signal transduction [13]. Furthermore, the KCa3.1 channel regulates membrane potential, cell volume, and Ca^2+^ signal transduction and is associated with oncogenic processes such as tumor formation, metastasis, and treatment resistance [14].

In addition, ionic balance in the tumor microenvironment plays an important role in regulating immune responses. This process, which is particularly effective in the migration and infiltration of T cells into tumor tissue, reveals the multifaceted roles of ions in cancer progression and treatment [15,16].

In general, all cells have a membrane potential between their internal and external environments, and this potential is largely determined by the activity of ion channels [10]. Thus, changes in membrane potential are frequently observed in cancer cells, and this contributes to the formation of the characteristic phenotypic features of malignant cells [1].

### 2.2. The Role of Ion Channels in Tumor Cell Behavior

Beyond being structures that regulate ion flow, ion channels are regulators that play a critical role in controlling fundamental biological processes of cancer, such as programmed cell death and metastatic behavior. Abnormal regulation of these channels in cancer cells has been associated with various cancer types [17,18].

In this context, the roles of ion channels in cancer cell behavior are evaluated within the framework of fundamental processes such as proliferation, apoptosis, metastasis, migration, and invasion.

#### 2.2.1. Role in Proliferation

Cell proliferation is a key element of cancer progression, manifesting as uncontrolled growth and division. Potassium channels have been shown to be highly expressed in various tumor cells and play a role in modulating cellular proliferation [19]. HERG (Kv11.1) activity has been shown to support the proliferation of cancer cells via TNF-α-mediated signaling pathways. In addition, studies have indicated that delayed rectifier K^+^ channels are effective in regulating cell cycle progression in different cell types [20,21].

Potassium channels and their subtypes play regulatory roles in different phases of the cell cycle, depending on the cell type and physiological state in which they are located. Voltage-dependent K^+^ channels, such as Kv1.3 and Kv1.5, contribute to the transmission of proliferative signals associated with the G1 phase of the cell cycle [22].

KCa3.1 (Ca^2+^-activated potassium channel) channels play a critical role in cell proliferation by regulating the membrane potential and intracellular Ca^2+^ levels. Numerous studies have reported that increased channel expression is associated with increased proliferative activity in cancer cells [23]. KCa3.1 channel expression increases cell proliferation by triggering p21 and p27 degradation via SPK2 activation [24]. KCa1.1 channel expression levels were positively correlated with the degree of malignancy. Pharmacological inhibition of these channels has led to S-phase arrest and cell death in glioma cells [25]. The Kir4.1 (inwardly rectifying potassium channel 4.1) potassium channel plays an important role in cell growth [3]. In glioma cells with deficient channel expression, the re-establishment of this channel causes a shift in the cell cycle from the G2/M phase to the quiescent G0/G1 phase, resulting in a significant decrease in growth capacity. In contrast, pharmacological inhibition of this channel reversed these effects. These findings show that Kir4.1 plays an important role in cell growth and maturation through its membrane hyperpolarization effect [26].

The effects of ion channels on cancer cell proliferation are related not only to their influence on membrane potential but also to the activation of signaling pathways that support tumor progression. Nav1.5 (voltage-gated sodium channel) has been reported to be expressed at increased levels in oral squamous cell carcinoma (OSCC) and to affect its proliferation. It has been reported that suppression of Nav1.5 leads to a decrease in cell proliferation, which may be related to the Wnt/β-catenin signaling pathway [27]. In another study, overexpression of the sodium channel subunit SCNN1B suppressed cell proliferation by inducing cell cycle arrest [28]. Inhibition of epithelial sodium channels (ENaC) led to cell cycle arrest in the G0/G1 phase in glioma cells. This effect was associated with increased expression of cyclin-dependent kinase inhibitor proteins p21 and p27 and inhibition of the ERK1/2 signaling pathway [29]. These findings suggest that sodium ion channels are crucial for regulating cancer cell proliferation.

Ca^2+^ controls various essential cellular processes, such as proliferation, differentiation, and gene transcription. Many studies have reported that some Ca^2+^ channels or pumps are downregulated or upregulated in several cancer types [30]. In addition, calcium ion channels play an active role in signal transduction during cell proliferation [31]. Studies have supported the idea that increased expression of L-type Ca^2+^ channels may regulate cell proliferation via Ca^2+^ influx in cancer cells [32]. The channel subunit Cav3.2 has been reported to be highly expressed in glioblastoma cells, and its inhibition affects the AKT/mTOR signaling pathway, reducing proliferation [33].

Mitochondria regulate intracellular Ca^2+^ signaling and play a role in the regulation of intracellular Ca^2+^ levels mediated by the mitochondrial Ca^2+^ uniporter (MCU). High expression of RIPK1 in colorectal cancer cells has been shown to promote cell proliferation by increasing Ca^2+^ uptake into the mitochondria through interaction with the mitochondrial Ca^2+^ uniporter (MCU) [34].

Chloride channels are transmembrane proteins that play an important role in the regulation of the cell cycle and proliferation, and studies related to their expression in cancer cells have been conducted [35]. Inhibition of the intracellular Cl^−^ channel (CLIC1) activates the JNK signaling pathway by increasing intracellular Ca^2+^ levels and ROS production in lung cancer cells. Therefore, it can be said that it has a regulatory effect on cancer cell viability through these processes [36]. However, the calcium-activated chloride channel ANO1 (TMEM16A) has been shown to regulate cell proliferation through different kinase activations and signaling pathways [37]. In addition, it has been reported that silencing CIC3 channels reduces chloride currents in glioma cells, which affects the cell cycle and reduces proliferation [38].

In addition, TRP (transient receptor potential) channels are associated with cell proliferation in various cancers. In non-small cell lung carcinoma cell lines, TRPC1 inhibition causes cell cycle arrest in the G0/G1 phase. This suggests that TRPC1 regulates EGFR (epidermal growth factor receptor)-mediated proliferation through calcium influx-dependent mechanisms [39]. In colorectal cancer, TRPC1 has been shown to increase proliferation and promote tumor development by activating the PI3K/AKT signaling pathway [40]. Silencing of TRPC1 reduces pancreatic ductal adenocarcinoma cell proliferation, and this effect is regulated by its interaction with PI3K-p85α and CaM [41].

These findings demonstrate that ion channels not only control electrical conduction but also play a critical role in the regulation of the cell cycle and growth signals.

#### 2.2.2. Role in Apoptosis

Apoptosis is a complex and sophisticated process involving many molecular mechanisms. Many of the morphological and biochemical changes observed in apoptotic cells during this process are mediated by several cysteine proteases, which are part of a large family of proteins expressed as caspases [42].

Many studies have identified ion channels as key regulators of apoptotic cell death. The effect of ion channels on apoptotic cell death varies greatly depending on the type of ion channel, cell type, and relevant intracellular signaling pathways [43]. Many studies have shown that different ion channels play important roles in the regulation of basic cellular processes [17,44,45].

Uncontrolled cancer cell proliferation is associated with irregular cell cycle progression and the avoidance of apoptosis. In this context, potassium channels stand out as structures that play a role in the regulation of apoptosis. Accordingly, intracellular K^+^ levels play a critical role in the regulation of apoptotic processes. A decrease in intracellular potassium ion levels is an important mechanism that triggers apoptosis [46,47]. In different cell types, it has been shown that the expression of K^+^ channels triggers apoptotic cell death due to the decrease in intracellular potassium ion levels [48,49]. It has been shown that disrupted mitochondrial homeostasis increases intracellular Ca^2+^ levels, thus activating Ca^2+^-sensitive K^+^ channels (maxi-K). It has been shown to support apoptosis through the activation of caspases due to a decrease in intracellular K^+^ levels [50]. In addition, it has been reported that increased extracellular K^+^ concentration inhibits early apoptotic events such as caspase and cytochrome C release [51]. Furthermore, HERG channels have been shown to enhance hydrogen peroxide-induced apoptosis in cancer cells [52]. Moreover, voltage-dependent K^+^ (Kv) and Ca^2+^-activated K^+^ (KCa) channel activations have been reported to be effective in nitric oxide-induced apoptosis [53]. In addition, it has been concluded that the activation of K^+^ and Cl^−^ channels is associated with Ca^2+^ and protein kinase C (PKC)-dependent mechanisms in TNF-α-mediated apoptosis [54]. In addition, Kv1.3 channels have been reported to be expressed in the mitochondria or plasma membranes of many cancer cells. Kv1.3 channel expression in mitochondria is associated with apoptosis [47]. Mitochondrial-targeted suppression of Kv1.3 increases apoptosis in pancreatic adenocarcinoma by impairing the mitochondrial membrane potential, increasing ROS levels, and activating the p38-MAPK signaling pathway [55].

Calcium, a common intracellular second messenger, regulates essential biological processes, including proliferation, differentiation, migration, and apoptosis [43,56]. Changes in the expression and function of Ca^2+^ channels, which regulate cellular Ca2 influx, play a critical role in cancer progression [56]. Voltage-gated calcium channels are key mediators that facilitate Ca^2+^ influx into cells in response to membrane depolarization. They play important roles in regulating crucial physiological processes, such as gene transcription [57]. Many studies have found that calcium channels are associated with the regulation of apoptosis [56]. It has been reported that Bcl-2 reduces Ca^2+^ influx into PC12 cells via L-type calcium channels and consequently reduces mitochondrial Ca^2+^ accumulation, thus increasing the cells’ resistance to the apoptotic process [58]. In this context, it has been shown that the increase in intracellular calcium levels resulting from the stimulation of the low-voltage-activated Ca^2+^ channel by cytokines triggers programmed cell death [59]. In contrast, inhibition of T-type calcium channels triggers apoptosis via p53-dependent signaling pathways, and this process is associated with p38-MAPK activation [60]. It has also been reported that blocking T-type Ca^2+^ channels with pharmacological and/or gene-silencing-based approaches triggers apoptosis via caspase activation in malignant melanoma cells [61]. In glioblastoma cells, this effect induces apoptosis by inhibiting the mTORC2/Akt signaling pathway and caspase activation [62]. Given the effects of T-type Ca^2+^ channels on the regulation of processes related to cancer progression, they are suggested as potential target molecules for anticancer therapies [60].

It has been shown that the TRPC1 channel plays an active role in overall cell survival and that inhibition of this channel can increase apoptotic processes. Overexpression of TRPC1 prevents cytochrome c release and mitochondrial-mediated apoptosis by inhibiting the translocation of Bax into the mitochondria [63].

The expression of SCNN1B, a sodium channel subunit, is an independent prognostic factor in gastric cancer cells. At the molecular level, it has been shown to interact with GRP78, an endoplasmic reticulum chaperone, leading to its degradation and triggering the activation of the unfolded protein response (UPR) pathways. This process activates stress response pathways, such as PERK-ATF4-XBP1s and C/EBP, leading to increased caspase-dependent apoptosis [28]. This data also demonstrate that ion channel components are important regulators of intracellular stress responses.

#### 2.2.3. Effects on Metastasis, Migration, and Invasion

Metastasis is an important factor that complicates the treatment process and significantly reduces the survival time of patients. Intracellular Ca^2+^ levels are a regulatory factor for migration and invasion, playing an important role in metastasis. It also regulates the Ca^2+^ driving force by activating Ca^2+^-dependent K^+^ channels [64].

Potassium channels and their subtypes have been shown to have a decisive effect on the regulation of proliferation and metastasis mechanisms in cancer cells. KCa3.1 expression has been reported to increase cellular migration and invasion by inducing epithelial–mesenchymal transformation (EMT) via reelin (RELN) [24]. It has been shown that KCa3.1 overexpression increases proliferation and migration in lung adenocarcinoma via AKT and ERK pathways, and that suppression of these pathways reduces these effects [65]. In addition, decreased KCNQ1 potassium channel expression has been reported in hepatocellular carcinoma, which is associated with poor prognosis. Studies have shown that KCNQ1 suppresses metastasis by inhibiting Wnt/β-catenin signaling [66].

Voltage-gated sodium channels (VGSC) are associated with cancer migration, invasion, and metastasis in some cancer types. Significantly increased Nav1.5 expression has been reported in metastatic cancer cells. Channel blockade with the VGSC blocker tetrodotoxin has been shown to significantly reduce cancer cell migration and invasion. Additionally, its correlation with ovarian cancer with lymph node metastasis suggests that voltage-gated sodium channels may be important factors in the metastatic spread [67]. Although the mechanisms underlying VGSC involvement in metastatic progression are not fully elucidated, factors such as protein interaction networks, changes in intracellular transport processes, and gene expression regulation have been proposed as possible mechanisms. High expression levels of Nav1.5 in colon cancer cells have been associated with poor prognosis and, when considered together with lymph node metastasis, have been reported as independent prognostic factors for lower disease-free survival [68]. In metastatic cells, sodium channel (Nav1.5) expression was higher than that in weak/non-metastatic cells [69]. This data also supports the idea that Nav1.5 is an effective factor in the metastatic processes of tumor cells. In oral squamous cell carcinoma, Nav1.5 has been shown to significantly inhibit the migration and invasion of cancer cells [27]. It has been emphasized that Nav1.6 and Nav1.7 expression are upregulated in prostate cancer cell lines, and this may be a diagnostic marker for some types of prostate cancer [70]. Another study reported that the Nav1.4 function is important for the invasive behavior of prostate cancer cells. A positive relationship between invasion capacity and channel expression was reported, and this Na^+^ channel expression was also increased in more invasive cell lines. Although the underlying molecular mechanism is not fully understood, it has been suggested that increased sodium channel expression may increase invasion by affecting Ca^2+^ homeostasis [71]. These data demonstrate that voltage-gated sodium channels are not only involved in ion transport but also important regulators of the invasive phenotype of tumor cells.

Abnormal expression of calcium channels plays an important role in cancer progression [72]. Excessive Cav1.3 expression has been observed in prostate cancer [73]. In addition, it has been reported that channel expression is positively correlated with metastasis and that inhibition of this channel suppresses AKT activity, reducing cell migration and invasion [74].

Transient receptor potential (TRP) ion channels also play a role in cancer progression. TRPC1 has been shown to play a role in the migration of glioma cells. Inhibition of this channel significantly reduces epidermal growth factor-mediated cell migration, and this effect has been reported to be related to calcium entry mechanisms [75]. In addition, increased TRPM8 expression has been reported to be associated with prostate cancer progression [76]. Conversely, some studies suggest that TRPM8 may have inhibitory effects on cell migration and angiogenesis [77].

In this context, Cl^−^ channels also play a role in regulating fundamental cellular functions, such as migration, cell volume homeostasis, and cell proliferation [3]. As a result of ion upregulation and associated water loss, cancer cells undergo changes in volume and shape, which support their migration and invasion [78]. Increased expression of ClC-2 and ClC-3 channels in glioma cells supports invasion by regulating changes in cell shape and size [79]. In addition, it has been reported that Cl^−^ channels (ANO1 and CLIC4) are upregulated in different cell types and are associated with cancer progression [80,81]. TMEM16A expression is increased in colorectal cancer cell lines with metastatic potential. Silencing this channel suppresses cell migration and invasion. This effect has also been reported to affect cellular motility and invasion processes in relation to MEK and ERK1/2 activation and decreased cyclin D1 expression [82].

In summary, diverse ion channel families regulate fundamental cellular processes in cancer cells, including proliferation, apoptosis, migration, and invasion, through a variety of intracellular signaling mechanisms. Depending on the channel type, distinct molecular pathways may be influenced, thereby contributing to the regulation of cellular events associated with tumor progression. These observations suggest that ion channels not only participate in ion transport but also serve as important regulatory elements involved in shaping cancer progression.

## 3. Ion Channel Modulators and Their Functional Effects on Cancer

**Figure 1:**
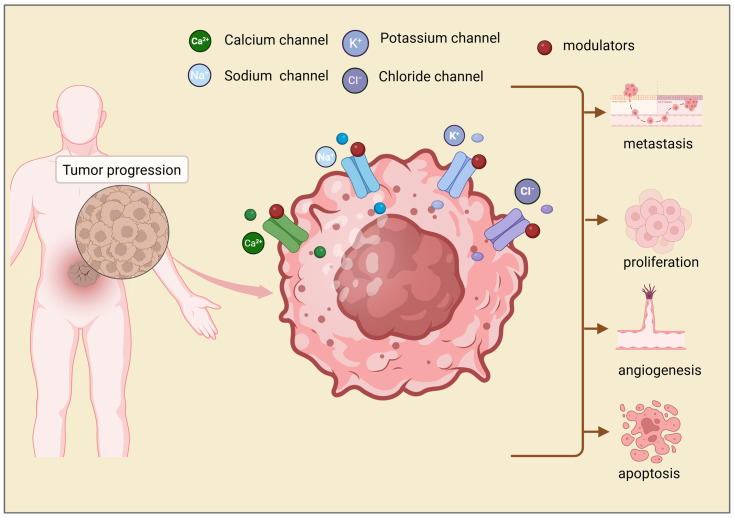
Ion channel modulators play a crucial role in modulating cellular processes associated with cancer. Created in BioRender. https://BioRender.com/l70645e (accessed on 25 May 2026).

Studies have shown that different ion channel modulators affect key processes, such as proliferation, cell migration, and invasion, in various cancer cell lines. As shown in Figure 1, ion channel activators or inhibitors modulate cellular processes, including tumor cell proliferation, migration, and invasion. Depending on the type of channel they target, these modulators have various functional effects on ion homeostasis, membrane potential, and intracellular signaling pathways. In particular, Na^+^, K^+^, Ca^2+^, and Cl^−^ channel modulators and their use and efficacy in cancer progression have been evaluated in this review. Representative studies on the use of ion channel modulators in different cancer models are summarized in Table 1.

### 3.1. K^+^ Channel Modulators

The roles of potassium channels in tumor cell progression are well-defined, and when these channels are blocked with pharmacological agents, anticancer effects have been observed. Indeed, suppression of the KCa3.1 channel with TRAM-34 or clotrimazole has been shown to reduce triple-negative breast cancer cell proliferation and migration while promoting apoptosis [83]. Another study also indicated a decrease in HepG2 cell proliferation with inhibition of these channels using TRAM-34 [84]. This data suggests that KCa3.1 channel blockers can be considered therapeutic targets. In addition, it has been reported that channel blockade suppresses prostate cancer cell proliferation, whereas KCa3.1 activation increases TRPV6-mediated Ca^2+^ influx through membrane hyperpolarization. In this context, KCa3.1 channel activity appears to support cell proliferation by regulating this influx [85].

Furthermore, Kv1.3 has been reported to respond to blockers such as dequalinium, amiodarone, and glibenclamide, inducing apoptosis by reducing growth [86]. In addition, it is targetable with pharmacological agents such as clofazimine, Psora-4, and PAP-1 [87]. K^+^ channel blockers such as tetraethylammonium, 4-aminopyridine, and verapamil have been reported to regulate tumor cell behavior in melanoma by suppressing the interaction between Kv1.3 potassium channels and β1 integrin [88].

Another K^+^ channel studied in the field of cancer is Eag (Kv10.1). The Eag blocker imipramine has been reported to suppress proliferation and increase apoptosis in ovarian cancer cells [89]. The hERG1 (Kv11.1) channel has also been reported to be significantly overexpressed in various cancer tissues [90,91]. In addition to its anticancer functions, tamoxifen has been shown to suppress Kv11.1 channel function [92]. It has also been reported that the use of some K^+^ channel inhibitors can cause serious cardiovascular side effects [93]. It is stated that this situation can be improved by targeting channel-related signaling complexes with modulators.

In addition, potassium channel activators can exhibit anticancer potential by affecting relevant cellular processes in malignant cells. Kv11.1 channel activation has been reported to suppress tumor growth in vitro and in vivo breast cancer cells by causing DNA damage and senescence [94]. Furthermore, it has been reported that NS1643 inhibits proliferation in breast adenocarcinoma by increasing p21 and p16INK4a protein levels, developing a senescence-like phenotype, and causing a G0/G1 phase arrest [95]. In another study, it was reported that activation of this channel increased oxidative stress in breast cancer. It has also been shown that suppression of the Nrf2-mediated antioxidant defense mechanism increases Kv11.1 channel activator-induced cell death [96].

It has been reported that KCNQ1 (Kv7.1) exerts a tumor suppressor effect in colorectal cancer by regulating the Wnt/β-catenin pathway. When pharmacologically suppressed, it has been shown to cause membrane depolarization and increased proliferation [97].

Many studies have suggested that potassium channels play an active role in cell cycle processes. It has been reported that suppression of the KCa3.1 channel with clotrimazole and TRAM-34 increases the accumulation of endometrial cancer cells in the G0/G1 phase [98]. In contrast, it has been reported that the use of the inhibitor (TRAM-34) halts cell cycle progression in LoVo cells at the G2/M phase [99]. This suggests that reducing KCa3.1 channel activity can halt the cell cycle in various phases and that this effect may vary depending on the cell type of the affected tissue. Therefore, channel inhibitors are potential candidates for cancer therapy. Inhibition of KCa3.1 (senicapoc) increases the adhesion of A549 lung cancer cells to microvascular endothelial cells due to increased ICAM-1 expression; however, it suppresses transendothelial migration. These findings suggest that the KCa3.1 channel plays a crucial role in the transmigration step of the metastatic cascade by regulating ICAM-1-dependent cell–cell interactions [100].

Two-pore domain potassium channels (K2P) are overexpressed in breast, leukemia, and lung cancer, whereas some subtypes are underexpressed in other cancer types. These findings suggest that ion channels may have multifaceted roles in cancer [101]. The application of a monoclonal antibody (Y4) targeting K2P9.1 has been reported to suppress metastasis growth in mice [102].

Increased KCa3.1 expression has been reported in colorectal cancer, particularly in cancers with KRAS mutations. Channel inhibition with caribdotoxin has been reported to reduce cell migration by suppressing Ca^2+^ influx and ROS-related signals. These findings suggest that these ion channels may be important regulators of the metastatic process [64].

Pharmacological modulation of ion channels is a strategy that can increase sensitivity to anticancer agents. Combination therapy with a KCa3.1 activator (SKA-31) and Kv11.1 inhibitor (E4031) or riluzole, which has both effects, increases cisplatin sensitivity by increasing apoptosis in colorectal cancer cells [103]. In addition, the potassium channel agonist minoxidil and the Na^+^ channel inhibitor ranolazine reduced dose-dependent cell invasion in triple-negative breast cancer cells. The combined use of ion channel modulators is also considered a potential anti-metastatic strategy [104].

### 3.2. Ca^2+^ Channel Modulators

Ca^2+^ channels have been reported to have different expression levels in different cancer types [105,106]. Therefore, repurposing pharmacological agents for their anticancer properties has been suggested to be beneficial [107]. Suppression of Cav1.3 in Hec-1A cells using nifedipine significantly reduced proliferation and migration [108]. It has been reported that T-type Ca^2+^ channel blockers (mibefradil and NNC-55-0396) inhibit cell proliferation in leukemia cells by halting the G1/S phase transition and trigger apoptosis partly due to endoplasmic reticulum-derived Ca^2+^ release [109]. In addition, it has been reported that nifedipine affects colorectal cancer and cancer immunity by inhibiting the NFAT2 signaling pathway [72]. It has been reported that the 3,4-dihydroquinazoline derivative (KYS05090), a T-type Ca^2+^ channel blocker, exhibits anticancer effects in mice bearing A549 xenografts and produces analgesic effects at higher doses [110]. In addition, both L- and T-type Ca^2+^ channel blockers have been reported to produce significant cytotoxic effects in HT-29 colon cancer cells [111].

Stimulation of Ca^2+^ channels affects the proliferative and metabolic activities of cancer cells [112]. The KYS05090 channel inhibitor has been shown to trigger autophagy and apoptosis by increasing ROS production in lung adenocarcinoma cells [113].

The combined use of Ca^2+^ channel modulators and anticancer drugs increases the sensitivity of cancer cells to these drugs. It has also been reported that the combination of amlodipine and lercanidipine blockers, which are Ca^2+^ channel inhibitors, with doxorubicin increases doxorubicin sensitivity in cancer cells and inhibits cell proliferation [114].

In conclusion, Ca^2+^ channel modulation affects pathways associated with tumor development and cell death. Modulating abnormal Ca^2+^ channel activity in cancer cells can disrupt cellular Ca^2+^ homeostasis, thereby influencing cancer progression.

### 3.3. Na^+^ Channel Modulators

The association of voltage-gated Na^+^ channels (VGSCs) with cancer cell invasion and metastasis has made the pharmacological modulation of these channels a potential therapeutic target. Phenytoin is one of the commonly used local anesthetics in clinical practice and acts by inhibiting voltage-gated sodium channels. Phenytoin has been reported to reduce migration and invasion in metastatic breast cancer cells, particularly in relation to Nav1.5 expression [115]. Similarly, lidocaine has been reported to suppress metastasis in ovarian cancer cells by inhibiting Nav1.5-mediated EMT and the FAK/paxil signaling pathway and to suppress postoperative pulmonary metastasis in a murine breast cancer surgical model [116,117]. Ropivacaine induces sorafenib-induced apoptosis in hepatocarcinoma cells by inhibiting the IL-6/STAT3 signaling pathway [118]. In addition, studies on lidocaine and bupivacaine in different cell lines are also available [119]. In addition, it has been shown that the VGSC channel activator veratrin increases proliferation in prostate cancer cells, while the blockers flunarizine and riluzole have the opposite effect [120]. In addition to these findings, similar results have been reported in studies using the channel inhibitors phenytoin and carbamazepine [121].

The potential of Na^+^ channel modulators to enhance the effects of existing treatments is noteworthy. For example, it has been reported that lidocaine, with Nav1.5 channel blockade, increases the sensitivity of ovarian cancer cells to carboplatin, paclitaxel [122], and cisplatin [116] based chemotherapies. In addition, it has been shown that lidocaine restores sensitivity in TMZ-resistant cells and reduces cell migration by inhibiting the HGF/MET signaling pathway in glioblastoma cells [123]. In addition, the riluzol-mFOLFOX6/bevacizumab combination has been reported to be well tolerated in metastatic colorectal cancer [124]. Furthermore, a reduction in lung metastasis has been reported when perioperative lidocaine is administered in combination with sevoflurane anesthesia in a breast cancer model [125].

Many peptide toxin modulators targeting Na^+^ channels have been investigated in experimental studies. In this context, VGSC inhibition with tetrodotoxin has been shown to reduce metastasis and significantly increase survival in vivo in prostate cancer [126]. Nav1.5 expression in colon cancer has been shown to increase the invasive effect of cancer cells by affecting invasion-related gene expression channels, and its suppression with tetrodotoxin has been shown to reduce invasive behavior [127]. It has also been reported to reduce cell invasion and migration in different types of cancer cells [69,128]. Modulation of Nav1.7 regulates the migration and invasion of JZTX-I/HNTX-III prostate cancer cells via Rho GTPase pathways [129].

### 3.4. Cl^−^ Channel Modulators

Voltage-gated Cl^−^ channels affect tumor processes in cancer cells. It has been reported that voltage-gated Cl^−^ channels ClC-2 and ClC-3 are upregulated in glioma cells, supporting cell proliferation and invasive cell migration [79,130]. It has been reported that CIC-3-related Cl^−^ channels can be inhibited by tamoxifen, 5-nitro-2-3-phenylpropylaminobenzoic acid (NPPB), and 4,4′-diisothiocyanatostilben-2,2′-disulfonic acid disodium salt hydrate (DIDS) in nasopharyngeal carcinoma cells [131].

It has been observed that the expression of multiple CIC channels in glioma cells is associated with invasion, and it has been reported that pharmacological suppression of CIC-3 reduces invasion, but invasion is more strongly inhibited with the non-specific ClC blocker NPPB [78]. These findings suggest that different Cl^−^ channels play a role in glioma cell invasion and that blocking multiple Cl^−^ channels may be more effective for treatment.

Changes in the expression or activity of Cl^−^ channels in various cancer types are associated with cancer progression. Bufalin, in addition to its Na^+^/K^+^-ATPase inhibitor properties, has also been reported to act as a CIC-3 channel activator, activating the CIC-3 Cl^−^ channel, inhibiting the PI3K/Akt/mTOR signaling pathway, and triggering apoptosis. Furthermore, the Cl^−^ channel blocker NPPB reduced these effects [132]. Similarly, lubiprostone has been reported to suppress tumor growth in colon cancer cells [133]. In addition, the CIC-3 channel activator bufalin has been reported to inhibit tumor microenvironment-mediated angiogenesis by suppressing the STAT3 signaling pathway in vascular endothelial cells [134]. Furthermore, chlorotoxin-containing fusion proteins suppress concentration-dependent cell migration in pancreatic cancer cells (PANC-1) by targeting chloride channel-related membrane proteins [135].

Chloride channels have also been shown to play a critical role in cell proliferation and the cell cycle. Cl^−^ channel blockers NPPB, niflumic acid (NFA), and tamoxifen have been reported to suppress proliferation and inhibit the G1/S transition in ovarian cancer cells [35]. It has been reported that this effect may be related to a Ca^2+^-dependent mechanism.

In conclusion, ion channels possess multifaceted and complex biological functions in tumor cells. The altered expression of these channels plays a significant role in cancer progression. Abnormal ion channel expression levels are associated with processes such as tumor cell proliferation, survival, and TME remodeling. In particular, the overexpression of certain ion channels can promote tumor cell growth and survival. Furthermore, ion channels can exert their effects on cancer through various signaling mechanisms. These data provide an important basis for evaluating ion channels as potential therapeutic targets.

**Table 1:** Ion channel modulators and their biological effects on cancer.

Types of Ion Channels	Modulator	Effect Type	Cancer Model	Biological Effect	Ref.
Cl^−^ channel	NPPB	Inhibitor	Ovarian cancer	Proliferation ↓	[35]
Cl^−^ channel	Niflumic acid	Inhibitor	Ovarian cancer	Proliferation ↓	[35]
L-type Ca^2+^ channel	Nifedipine	Inhibitor	Colorectal cancer	Proliferation ↓ Metastasis ↓	[72]
Cl^−^ channel	NPPB	Inhibitor	Glioma	Invasion ↓	[78]
KCa3.1	TRAM-34	Inhibitor	Triple-negative breast cancer	Proliferation ↓, Migration ↓, Apoptosis ↑	[83]
KCa3.1	Clotrimazole	Inhibitor	Triple-negative breast cancer	Proliferation ↓, Migration ↓, Apoptosis ↑	[83]
KCa3.1	TRAM-34	Inhibitor	Hepatocellular carcinoma	Proliferation ↓	[84]
Kv1.3	Dequalinium	Inhibitor	Prostate cancer	Apoptosis ↑	[86]
Kv1.3	Amiodarone	Inhibitor	Prostate cancer	Apoptosis ↑	[86]
Kv1.3	Glibenclamide	Inhibitor	Prostate cancer	Apoptosis ↑	[86]
Kv10.1	İmipramine	Inhibitor	Ovarian cancer	Proliferation ↓ Apoptosis ↑	[89]
Kv11.1	NS1643	Activator	Breast cancer	Tumor growth ↓	[94]
Kv11.1	NS1643	Activator	Breast cancer	Proliferation ↓, Senescence induction ↑	[95]
Kv7.1	Chromanol 293B	Inhibitor	Colorectal cancer	Proliferation ↑, EMT ↑	[97]
KCa3.1	Senicapoc	Inhibitor	Lung cancer	Cell adhesion ↑, Migration ↓	[100]
KCa3.1/Kv11.1	Riluzole	Activator/ Inhibitor	Colorectal cancer	Apoptosis ↑ Cisplatin efficacy ↑	[103]
K_ATP_ VGSC	Minoxidil + Ranolazine	Activator Inhibitor	Breast cancer	Invasion ↓	[104]
L-type Ca^2+^ Channel	Nifedipine	Inhibitor	Endometrial carcinoma	Proliferation ↓ Migration ↓	[108]
T-type Ca^2+^ Channel	Mibefradil	Inhibitor	Leukemia	Proliferation ↓ Apoptosis ↑	[109]
T-type Ca^2+^ Channel	NNC-55-0396	Inhibitor	Leukemia	Proliferation ↓ Apoptosis ↑	[109]
T-type Ca^2+^ Channel	KYS05090	Inhibitor	Lung cancer	Antitumor ↑ Tumor growth ↓	[110]
T-type Ca^2+^ Channel	NNC-55-0396	Inhibitor	Colon cancer	Cytotoxicity ↑	[111]
L-type Ca^2+^ Channel	Amlodipine	Inhibitor	Colon cancer	Cytotoxicity ↑	[111]
L-type Ca^2+^ Channel	Verapamil	Inhibitor	Osteosarcoma	Proliferation ↓	[112]
T-type Ca^2+^ Channel	KYS05090	Inhibitor	Lung adenocarcinoma	Autophagy ↑ Apoptosis ↑	[113]
L-type Ca^2+^ Channel	Lercanidipine	Inhibitor	Gastric cancer	Proliferation ↓, Doxorubicin Efficacy ↑	[114]
L-type Ca^2+^ channel	Amlodipine	Inhibitor	Gastric cancer	Proliferation ↓, Doxorubicin Efficacy ↑	[114]
Nav1.5	Phenytoin	Inhibitor	Breast cancer	Migration ↓, Invasion ↓	[115]
Nav1.5	Lidocaine	Inhibitor	Ovarian cancer	EMT ↓, Metastasis ↓,	[116]
Na^+^ channel	Ropivacaine	Inhibitor	Hepatocellular carcinoma	Cisplatin efficacy ↑ Proliferation ↓, Apoptosis ↑	[118]
Na^+^ channel	Bupivacaine	Inhibitor	Breast cancer	Apoptosis ↑	[119]
Na^+^ channel	Lidocaine	Inhibitor	Breast cancer	Apoptosis ↑	[119]
VGSC	Veratrine	Activator	Prostate cancer	Proliferation ↑	[120]
VGSC	Flunarizine	Inhibitor	Prostate cancer	Proliferation ↓	[120]
VGSC	Riluzole	Inhibitor	Prostate cancer	Proliferation ↓	[120]
VGSC	Phenytoin	Inhibitor	Prostate cancer	Proliferation ↓	[121]
VGSC	Carbamazepine	Inhibitor	Prostate cancer	Proliferation ↓	[121]
VGSC	Lidocaine	Inhibitor	Epithelial ovarian	Proliferation ↓	[122]
VGSC	Bupivacaine	Inhibitor	Epithelial ovarian	Proliferation ↓	[122]
VGSC	Lidocaine	Inhibitor	Glioblastoma	Migration ↓	[123]
Na^+^ channel	Lidocaine	Inhibitor	4T1 murine model of breast cancer	Metastasis ↓, Angiogenesis ↓	[125]
VGSC	Tetrodotoxin	Inhibitor	Prostate cancer	Metastasis ↓ Survival ↑	[126]
Nav1.5	Tetrodotoxin	Inhibitor	Colon cancer	Invasion ↓	[127]
VGSC	Tetrodotoxin	Inhibitor	Lung cancer	Invasion ↓	[128]
Nav1.7	JZTX-I	Activator	Prostate cancer	Migration ↑, Invasion ↑	[129]
Nav1.7	HNTX-III	Inhibitor	Prostate cancer	Migration ↓ Invasion ↓	[129]
CIC-3 Cl^−^ channel	Bufalin	Activator	Hepatocellular carcinoma	Apoptosis ↑	[132]
CIC-2 Cl^−^ channel	Lubiprostone	Activator	Colon cancer	Tumor growth ↓	[133]
CIC-3 Cl^−^ channel	Chlorotoxin	Inhibitor	Pancreatic cancer	Migration ↓	[135]

Arrows indicate the following: ↑, upregulation; ↓, downregulation.

## 4. Limitations in the Traditional Use of Ion Channel Modulators

The cancer microenvironment is highly complex. Owing to its heterogeneous structure, the therapeutic effects of conventional treatments may be limited. Ion channels play important roles in tumor processes, such as proliferation, apoptosis, cell migration, and invasion [45]. Therefore, pharmacological targeting of these channels is a promising strategy for anticancer treatment. Although ion channel modulators have therapeutic potential in cancer treatment, their use is limited by several challenges and limitations.

Selectivity is one of the most fundamental limiting factors. Specific targeting is a major challenge because ion channels are widely expressed in almost every tissue. Therefore, the use of ion channel modulators can cause significant side effects in the body. Off-target toxicity, such as arrhythmia [93,136], is a significant problem. In addition, ion channel modulators have limitations, such as exhibiting limited pharmacokinetic properties and not reaching effective concentrations in cancer tissue owing to their short circulation time [23,137]. Although specific modulators have been developed, the structural conservatism between channel subunits makes specific targeting difficult.

Furthermore, traditional in vitro cell culture systems cannot adequately mimic the cancer microenvironment components that affect ion channel activity, such as hypoxic conditions and cell–cell interactions. In addition, the lack of preclinical models that accurately reflect ion channel biology and cancer-host relationships limits the clinical relevance of efficacy and toxicity analysis.

These limitations have increased interest in drug delivery systems that can enhance therapeutic efficacy and reduce systemic side effects of ion channel modulators (inhibitors and activators). Hydrogel-based drug delivery systems are considered a promising approach owing to their ability to provide localized and controlled drug release and to respond to the tumor microenvironment. In addition, many ion channel modulators suffer from rapid clearance and limited accumulation at tumor sites, which may reduce their therapeutic efficacy. Hydrogel-based systems can improve drug retention at the target site while minimizing exposure to healthy tissues. Therefore, hydrogel-based platforms have attracted considerable attention for the delivery of ion channel modulators. Further details are discussed in Section 6.

## 5. Classification of Hydrogels Based on Structural Dimensions

**Figure 2:**
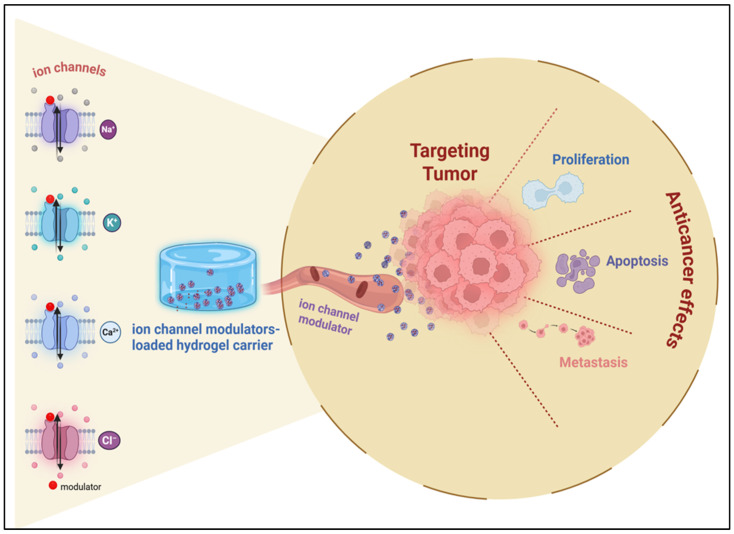
Schematic representation of the ion channel modulator-loaded hydrogel system and its anti-cancer effect. Created in BioRender. https://BioRender.com/xloo32k (accessed on 25 May 2026).

Gels are three-dimensional, cross-linked network structures in the liquid phase [138]. These structures have soft characteristics and consist of polymer chains stabilized by physical/chemical bonds [139]. Simultaneously, owing to their high liquid retention capacity and viscoelastic properties, gels have a wide range of applications in both scientific literature and applied fields. Hydrogels, which are important among gels, are defined as hydrophilic polymer networks that can swell in water or biological fluids [140]. Owing to their biocompatibility, soft tissue-like mechanical properties, and sensitivity to environmental stimuli, hydrogels are used in many biomedical applications, primarily in controlled drug delivery, tissue engineering, and biosensor applications [5]. Hydrogel systems developed over time have been reduced to micro and nano dimensions, providing a new perspective on hydrogels. In this context, structures smaller than 100 nm are generally defined as nanogels, and structures up to the micrometer scale are defined as microgels [141]. In addition, micro-and nanoscale structures are considered “smart materials” because of their ability to respond quickly to environmental stimuli [142]. These systems are high-performance, dynamic, soft, permeable, and deformation-adaptable. However, the fact that hydrogel systems at different scales are mostly studied independently in the literature leads to insufficient information regarding their dimensions and functional capabilities. This deficiency necessitates a holistic approach to address macro, micro, and nanogel systems. As illustrated in Figure 2, hydrogels can serve as carriers for ion channel modulators, enabling their localized and controlled delivery to tumor tissues. Following release from the hydrogel matrix, these modulators interact with cancer-associated ion channels, including Na^+^, K^+^, Ca^2+^, and Cl^−^ channels, that regulate key cellular processes. By modulating ion channel activity, hydrogel-based delivery systems may suppress tumor cell proliferation, induce apoptosis, and inhibit metastatic progression. Therefore, hydrogels not only improve the pharmacokinetic profile of ion channel modulators but also enhance their therapeutic impact on tumor-associated signaling pathways. In this context, the classification of hydrogels according to their dimensions is discussed in the following sections. Although macro-, micro-, and nanogel systems offer distinct advantages, each category also presents specific challenges that may affect its clinical translation. A comparison of the major advantages and clinical translation challenges associated with different hydrogel size categories is presented in Table 2.

### 5.1. Bulk (Macroscopic) Hydrogel Systems

Macrogels are generally classified as hydrogel systems capable of forming polymer networks with macroscopic dimensions exceeding 100 µm. Hydrogels are defined as systems with at least two components containing water molecules in the spaces between macromolecules [143]. The crosslinking density is generally used to ensure the mechanical integrity of these systems. This allows the macrogels to exhibit high structural stability. However, this dense and flexible network structure suggests that diffusion primarily provides a limited passage through intra-network pores and significantly restricts mass transfer. It is also known that the interaction of the gel with its environment is weakened when there is a low surface area or volume ratio [144]. This leads to macrogels exhibiting more functionality in extracellular environments than in intracellular applications. However, these limitations also present advantages for macrogels. Slow diffusion can provide a stable profile in systems requiring long-term and controlled release. In addition, their high mechanical strength makes them suitable for use as tissue engineering scaffolds and implant materials [145,146]. In conclusion, macrogels are increasingly preferred for applications requiring structural support and long-term function.

Polyurethane (PU) [147], poly(vinyl alcohol) (PVA/PVAL) [148], polyethylene glycol diacrylate (PEGDA) [149] and its derivatives, and poly(2-hydroxyethyl methacrylate) (PHEMA) [150] are commonly used in macrogel synthesis. In addition, macrogels used in biomedical applications are mostly obtained from natural polymers, particularly polysaccharide derivatives. Polysaccharides such as alginate, chitosan [151], pectin [152], and hyaluronic acid [153] are generally preferred in macrogel studies because of their biocompatibility, biodegradability, and ability to interact with cells.

Macrogels can be classified physically and chemically according to the type of crosslinking. Chemically cross-linked macrogels are formed by covalent bonds between polymer chains [154]. In this case, they generally exhibit high mechanical strength and structural stability. These types of macrogel systems are usually synthesized by methods such as radical polymerization, ultraviolet (UV) or ionizing radiation, or by using multifunctional cross-linking agents. In contrast, physically cross-linked macrogels are formed as a result of weak and reversible interactions, such as hydrogen bonds, ionic interactions, hydrophobic interactions, and chain entanglement [155]. Although these systems generally exhibit lower mechanical strength, they offer advantages such as stimulus sensitivity, self-healing properties, and biocompatibility. When evaluated in terms of drug delivery applications, the presence of pores in the macrogel structure is advantageous for macrogel systems. Studies have reported that the three-dimensional network structure in macrogels allows the loading of active components into the hydrogel matrix [156,157].

**Figure 3:**
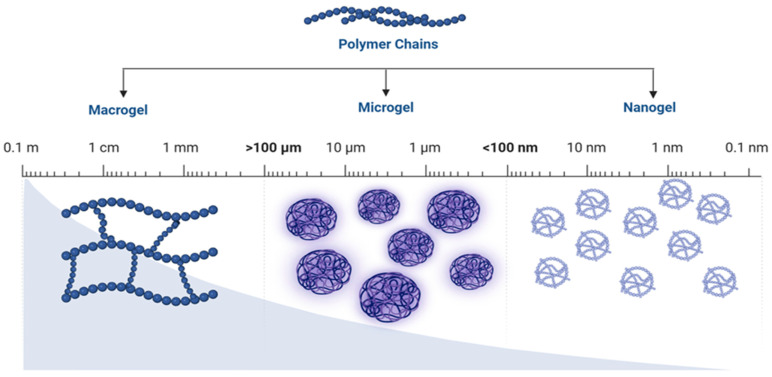
Structural classification of hydrogel systems with different size ranges. Created in BioRender. https://BioRender.com/nmfcel1 (accessed on 25 May 2026).

One of the most notable applications of macrogels in drug delivery systems is in cancer treatment [158]. Chemotherapy, widely used in cancer treatment today, is effective, but the drug reaching not only the tumor area but also healthy tissues can cause serious side effects [159]. Macrogels, developed to reduce this problem, can release drugs in a more controlled manner by targeting the acidic environment of the tumor, especially because of their pH-sensitive structure [160]. This approach offers a significant advantage by increasing the effectiveness of the treatment and reducing side effects. For example, Recent studies have further demonstrated the potential of hydrogel-based delivery systems to enhance the therapeutic efficacy of anticancer agents while reducing systemic toxicity. For example, a degradable and self-healing hydrogel platform co-loaded with ursolic acid and cisplatin significantly enhanced antitumor activity and reduced systemic toxicity in vivo compared with conventional treatment approaches. These findings highlight the advantages of localized and sustained drug delivery provided by hydrogel systems in cancer therapy [161]. In addition, it has been reported that the cytotoxic effect on cancer cells is increased with controlled drug release using HEMA/pectin-based carrier systems loaded with ion channel blockers [162]. ]. Figure 3 illustrates the size-based classification of hydrogels into macrogels, microgels, and nanogels. Differences in particle size directly influence drug loading capacity, release behavior, tissue penetration, and biodistribution. In general, macrogels are suitable for localized and sustained release, whereas microgels and nanogels offer improved injectability, cellular uptake, and tumor penetration. Therefore, hydrogel size is a critical parameter in the design of ion channel modulator delivery systems and may significantly affect their therapeutic performance.

However, some studies have aimed to add functionality to hydrogel materials by grafting different groups onto them [162]. Thus, the application areas of hydrogels can be further expanded in the future. In a recent study, an injectable, adhesive, and self-healing microgel-based hydrogel system was developed for the treatment of infected wounds. This microgel-based structure was observed to have antibacterial, Reactive Oxygen Species (ROS) clearing, and wound-healing accelerating properties [163]. The superior properties of these systems, stemming from their dynamic, permeable, and easily deformable structural properties, significantly contribute to maintaining stability, especially during the transport of biopharmaceutical molecules, such as proteins.

In addition, some structural and functional limitations of traditional hydrogel systems in clinical applications have necessitated the redesign of these materials at the dimensional scale. Problems such as the rapid release of hydrophilic drugs, low loading capacity for hydrophobic drugs, and large macroscopic size of hydrogels are major limiting factors in pharmaceutical drug delivery applications [164]. Thus, the development of hydrogel platforms at different scales, such as microgels and nanogels, has become necessary, especially in the biomedical field.

### 5.2. Microgels: Colloidal Hydrogel Particles

Microgels are located between macro-and nanoscale systems, with sizes ranging from approximately 100 nm to 100 µm [165]. This intermediate scale allows not only dimensional reduction but also rearrangement of the hydrogel network structure. In particular, a more balanced crosslinking density allows both the preservation of mechanical stability and an increase in swelling capacity [166,167].

The microscale polymeric network structure and large surface area provide exceptional permeability [168]. Thus, they enable efficient absorption and release of materials. They can also respond dynamically to external changes, swelling, or shrinking. This makes them excellent carriers for active substances, such as drugs, bioactive molecules, and biochemical signals [169]. Recent research has shown that when metallo-supramolecular crosslinkers are used instead of conventional *N*,*N*′-methylenebisacrylamide, poly(N-isopropyl acrylamide) (pNIPAm) microgels or other acrylamide derivatives with a volume phase transition temperature (VPTT) in pure water are concentrated [170,171].

Microgels are generally divided into two main groups (chemical and physical) depending on the crosslinking mechanism [172]. Chemical microgels are formed by covalent bonds between polymer chains. Such structures are mostly formed through cross-linking agents such as glutaraldehyde and epichlorohydrin or enzymatic reactions. In contrast, physical microgels are formed by noncovalent interactions, such as hydrogen bonds, hydrophobic interactions, and ionic or electrostatic forces. Although microgels have similar polymer chemistry and crosslinking mechanisms as macrogels, the main difference stems from their structural organization and size scale. Microgels exhibit faster diffusion and more sensitive responses to environmental stimuli owing to their discrete particle structures and high surface areas. In contrast, macrogels generally exhibit more limited dynamic behavior because of their continuous network structures. In cell encapsulation applications, the semi-permeable network structure of microgels allows the diffusion of nutrients and metabolites while providing a protective microenvironment for encapsulated cells [173].

A review of the current literature reveals that pNIPAm-based stimulus-responsive microgels have been extensively researched [174]. The rapid response of these structures to temperature changes has been emphasized. A recent study reported the development of a new microgel system exhibiting rapid swelling-shrinkage and dispersion-aggregation release behavior by increasing the VPTT value through the addition of acrylamide (AAm) comonomer to the system, thus preventing aggregation at higher temperatures [175]. This approach demonstrates the potential of smart hydrogel systems for fast-responding actuators and advanced biomaterial applications. In contrast, microgel-reinforced zwitterionic hydrogel coatings are promising materials that improve both mechanical strength and biocompatibility, particularly on device surfaces in contact with blood [176]. The microgel reinforcement approach has been shown to significantly overcome these limitations, improving both the mechanical strength and anti-swelling properties. This hybrid structure, achieved through microgel reinforcement, demonstrates that mechanical stability can be enhanced without sacrificing biocompatibility. Thus, it makes a significant contribution to next-generation surface coating strategies for biomedical devices that come into contact with blood.

In this respect, microgels offer a balance between the mechanical advantages of macrogels and the functional properties of nanogels, creating a broad platform for advanced biomedical applications. In this context, Moon et al. developed an injectable and biocompatible microgel-based system for controlled immune modulation. In this study, we combined the therapeutic potential of dendritic cells (DCs) with the versatility of alginate hydrogels. Stable crosslinking at room temperature was achieved by encapsulating microparticles within injectable alginate structures. In vitro results showed that encapsulated DCs could proliferate for up to 7 days, demonstrating that these systems have significant potential for long-term immune activation [177]. Recently, Chen et al. developed an innovative microgel-based system targeting the in vivo activation of the FGF18 gene for the treatment of osteoarthritis. In this study, the CRISPR/Cas9 system was delivered via chondrocyte-affinity peptide-modified hybrid exosomes and encapsulated in a methacrylic anhydride-modified hyaluronic acid-based microgel. This developed injectable microgel system has been shown to enhance cartilage regeneration, reduce inflammation, and suppress ECM degradation in both in vitro and in vivo studies [178].

Microgels are highly effective encapsulation systems for the protection and controlled release of bioactive compounds. For example, a study reported in the literature showed that polyphenols obtained from *Juglans regia* bark could be encapsulated using chitosan-and sodium alginate-based pH-sensitive microgels. The microgel structure increased the physicochemical stability of the compounds and was investigated in controlled release studies under gastrointestinal conditions. Thus, the degradation of polyphenols during digestion was significantly prevented, and their bioavailability could be increased by microgel encapsulation [179]. Although many studies on smart microgels have been conducted, in vivo safety and efficacy data to support the clinical applicability of these systems are still limited. Thus, the transition to nanogel systems has become inevitable in situations that require more advanced targeting and intracellular transport.

### 5.3. Nanogels: Nanoscale Functional Carriers

Nanogels are hydrogel systems that exhibit colloidal properties, with dimensions below 100 nm [180]. Similar to classical hydrogels, nanogels have a high water-holding capacity owing to the abundant hydrophilic groups and network structures in their composition [181]. Nanogels can be classified according to their sensitivity to stimuli, type of crosslinking (physical/chemical), composition (natural/synthetic/hybrid), structure and organization (micelle, liposome modified, etc.), and size, shape, and surface properties. Examples of nanogels made from synthetic polymers, such as polyethylene glycol, N-vinylcaprolactam [182], and poly(N-isopropylacrylamide) [183], or natural polymers, such as chitosan [184] and alginate [185], can be given. These structures are cross-linked chemically or physically through covalent or noncovalent bonds. Owing to these properties, the physicochemical properties of nanogels, such as their size, shape, surface charge, and mechanical strength, can be controlled.

**Figure 4:**
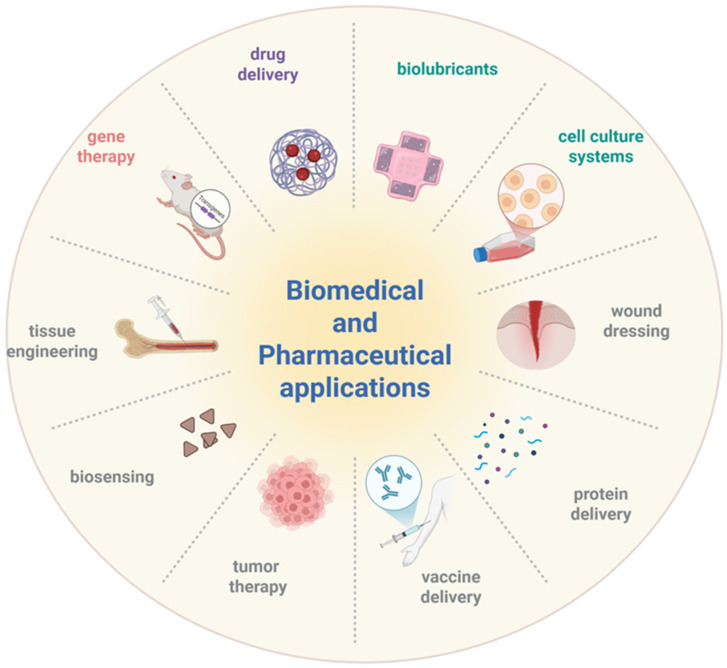
Schematic illustration of the main biomedical and pharmaceutical applications of macrogel, microgel, and nanogel systems. Created in BioRender. https://BioRender.com/mqhmffm. (accessed on 25 May 2026).

In contrast to macro- and micro-hydrogels, nanoscale hydrogels offer significant advantages in terms of bio-binding and encapsulation of bioactive substances by providing a high specific surface area and similarity to nanoparticles [186]. In addition, owing to their small size, they can overcome certain physiological barriers more effectively than macro-sized hydrogels. In particular, the large surface area and porous structure of nanogels offer a significant advantage in terms of their high drug-loading capacity and controlled drug release profile. When the literature is examined, it is seen that these systems have significant potential for use in numerous biomedical and pharmaceutical application areas, such as drug delivery, gene therapy, tissue engineering, wound healing, and tumor treatment, owing to their structural and functional properties. Figure 4 summarizes the various application areas of macrogel, microgel, and nanogel systems depending on their dimensions and structural properties.

In addition, nanogels have the potential to cross the blood–brain barrier (BBB). The BBB is a selective barrier that prevents most drugs from passing into the brain. Many studies have shown that nanogels have the potential to overcome this barrier because of their small size and ability to undergo surface modification [187,188]. In particular, some studies have shown that nanosized gels can cross the BBB via endocytosis by brain endothelial cells [189]. In addition, nanogels modified with ligands, such as peptides, antibodies, and hyaluronic acid, enable targeted drug delivery via receptor-mediated transport [190].

For example, Yue Hu et al. developed an HA-based nanogel system that is sensitive to reactive oxygen species (ROS) and is coated with exosomes. The developed nanogel system loaded with PACAP and estrogen (E2) showed high cellular uptake in vitro and in vivo and could cross the blood–brain barrier. These findings reveal that exosome-coated nanogels are promising drug delivery platforms [191].

Furthermore, soft nanogels can be considered an alternative platform to Gliadel wafers for traditional local anticancer drug delivery after brain tumor resection. Smart nanogels have been shown to have the potential to support axonal regeneration and restore motor function loss by targeting activated astrocytes in glial scarring after spinal cord injury [192]. These systems are not limited to neurological and oncological applications but also show potential in chronic wound treatment. Zhang et al. reported that a hydrogel-based nanogel accelerated wound healing by providing controlled growth factors and DNase I release in diabetic wound models [193].

In cases involving multistage pathological processes, such as spinal cord injury, hydrogel-based nanogel systems that provide sequential controlled drug release are of increasing interest. In this context, Huang et al. developed a smart hydrogel platform that enables the co-release of melatonin and ibuprofen to target different pathological stages of spinal cord injury [194]. This system has also been shown to be effective in suppressing inflammation and supporting axonal regeneration. Consequently, when comparing nanogels to macro-and microscale hydrogel systems, the advantages of improved surface area, cellular internalization, and controlled release make them a more functional platform for advanced biomaterial applications.

## 6. Delivery of Ion Channel Modulators via Hydrogel Systems

Although the effects of ion channel modulators in cancer treatment have been defined, studies on the targeted delivery of these modulators using hydrogel-based carrier systems appear to be limited. Research shows that these systems are promising in terms of increasing bioavailability by providing target-specific and controlled release. Studies on the delivery of ion channel modulators in hydrogel-based carrier systems are presented in Table 3.

Use of ion channel modulators is limited by systemic side effects and low bioavailability. In this context, injectable hydrogels developed to enhance the therapeutic efficacy of ion channel modulators are noteworthy. A chitosan/pluronic acid-based hydrogel system functionalized with the Kv11.1 potassium channel activator NS1643 was designed, and in vivo experiments, it was reported that tumor growth was significantly suppressed in a triple-negative breast cancer model. Furthermore, it was reported that no systemic toxicity was observed, and the drug showed local and controlled release. These results show that the application of ion channel activators with hydrogel systems can increase the therapeutic efficacy [195].

Hydrogel-based carriers, thanks to their three-dimensional structure, allow for the delivery of multiple therapeutic agents simultaneously or with controlled release [196]. The combined use of ion channel modulators and chemotherapeutic agents on these platforms allows for the development of combined treatment strategies. Natural-product-based hydrogels, including alginate, chitosan, gelatin, and hyaluronic acid, represent promising delivery platforms for ion channel modulators owing to their biocompatibility, biodegradability, and ability to provide sustained drug release toward cancer-associated targets. In this context, a hybrid NLC-hydrogel formulation was developed in which nanostructured lipid carriers (NLCs) loaded with docetaxel and lidocaine were integrated into a xanthan-chitosan-based hydrogel system. In vitro studies revealed that docetaxel showed lower cytotoxicity after encapsulation in the hybrid formulation, which was associated with sustained drug release. In vivo studies showed that the hybrid hydrogel could inhibit tumor growth, but was equivalent to free docetaxel treatment. Imaging data suggest that combined treatment strategies are associated with a better prognosis [197]. In this context, it has been reported that the delivery of lidocaine along with cisplatin to a nanogel system modified with cRGDfk peptide, which shows high affinity to αvβ3 integrin, contributes to the suppression of primary tumor growth and reduces side effects associated with chemotherapeutic agents [198]. In addition, the incorporation of ropivacaine into a cisplatin-loaded PF127 hydrogel increased MHC-I expression and T lymphocyte response, thereby enhancing antitumor response and chemotherapy efficacy [199]. This demonstrates that targeted delivery platforms can provide multifaceted advantages in cancer treatment.

The effectiveness of chemotherapeutic agents in cancer treatment is limited by multidrug resistance (MDR). Hydrogel-based delivery of ion channel modulators offers an effective approach to overcome MDR. In this context, the co-deployment of the calcium channel blocker verapamil and the chemotherapy agent doxorubicin via hydrogel nanoparticle systems has increased intracellular drug accumulation in tumor cells. This strategy enhances chemotherapeutic efficacy by suppressing drug efflux pumps and overcoming multidrug resistance in tumor cells [200]. Similarly, the co-deployment of verapamil and doxorubicin in stimulus-responsive peptide-based nanogel systems has been reported to increase intracellular drug accumulation in resistant cancer cells and provide significantly higher antitumor activity than free doxorubicin [201]. These findings demonstrate that the use of ion channel modulators in combination with hydrogel-based delivery systems offers a significant strategy for overcoming MDR.

Nanotechnology-based carrier systems offer significant advantages in enhancing the efficacy of ion channel modulators and reducing systemic toxicities. In this context, a study using potassium channel blocker-loaded cross-linked HEMA/pectin nanogels reported cytotoxicity in cancer cells compared with normal cells. However, the effect of nanoencapsulation varies depending on the drug; hormesis-like behavior was reduced with dofetilide but persisted with azimilide. In addition, it was shown that the combination with paclitaxel in cancer cells increased the cytotoxic effect only for dofetilide, while no change was detected for azimilide. These findings revealed that the therapeutic efficacy of ion channel modulators is related not only to the channel target but also to the drug combinations and carrier properties used [162].

Hydrogels are powerful systems that enable the controlled and efficient delivery of various pharmacological agents, making them promising for the targeted delivery of ion channel modulators in cancer therapy. It has been shown that diltiazem hydrochloride-loaded hydrogel microsphere formulations provide higher drug loading capacity and controlled release properties compared to normal hydrogels [202]. Similarly, it has been shown that various drug releases can be regulated in a controlled manner in different hydrogel systems [203,204,205,206]. In addition, pH-sensitive IPN hydrogel systems have been developed for the controlled release of the Ca^2+^ channel blocker verapamil, and it has been shown that drug release is dependent on pH and polymer composition [207].

Furthermore, hydrogels are among the prominent platforms for the controlled delivery of therapeutic agents because of their high-water content, biocompatibility, and structural properties similar to soft tissues. In this regard, a copolymeric hydrogel system loaded with sodium channel blockers has been reported to show high biocompatibility, suppress in vivo tumor growth, and reduce toxicity [208]. Hydrogel-based carriers have been reported to enhance therapeutic efficacy through controlled drug release and by increasing the bioavailability of low-solubility drugs [209]. Studies have also supported the applicability of this approach to ion channel modulators. For example, the delivery of nifedipine via a HEMA hydrogel system has demonstrated biological efficacy through controlled drug release [210].

The functionality of ion channels in the tumor microenvironment highlights their importance as therapeutic targets for cancer treatment. Nervous system components found in the tumor microenvironment have been shown to contribute to cancer pathogenesis [211], and pharmacologically targeting this interaction is a promising therapeutic approach. In this context, it has been reported that suppressing neuron-cancer communication using 100 nm size PEG-plated lipid nanoparticles loaded with bupivacaine, a Na^+^ channel blocker, reduces tumor growth and metastatic spread [212].

In recent years, hydrogel-based local drug delivery systems have emerged as a promising approach for the postoperative management of tumors because of their ability to deliver high drug concentrations and to provide controlled, long-term release profiles [213]. In the study presented by Fu et al., it was observed that tumor growth was suppressed as a result of the co-administration of STAT3 siRNA and lidocaine with an injectable alginate-based hybrid hydrogel. The controlled release of lidocaine from this carrier platform contributed to the antitumor effect by increasing natural killer cell activation [214].

In another study, a hydrogel system containing bupivacaine-loaded nanocapsules provided extended and controlled drug release. Although these hydrogel platforms have been applied in studies such as pain management, the advantages of controlled release and biocompatibility offer significant potential for the targeted delivery of ion channel modulators [215].

Naturally derived compounds are promising because they can regulate cancer cell progression by acting on ion channels. Although studies on the delivery of these agents using hydrogel-based carriers are limited, promising results have been reported. For example, naturally derived matairesinoside (MTS) has been shown to inhibit the TMEM16A calcium-activated chloride channel, which has been loaded into a biodegradable and self-healing functional hydrogel system to create a targeted platform. In vivo experiments have shown that the hydrogel/MTS formulation effectively suppresses tumor growth in lung cancer. This supports the idea that the hydrogel system enhances the antitumor properties of MTS through sustained release. Furthermore, it has been reported that the side effects of free drug administration are significantly reduced with this formulation [216]. Similarly, it has been reported that tumor growth is inhibited and toxicity is reduced with a limonin-loaded hydrogel system targeting the TMEM16A ion channel [217]. In addition, silibinin-loaded biodegradable Pec-H/DCMC hydrogels targeting the same ion channel have been reported to enhance in vivo antitumor efficacy and reduce toxicity [218]. The delivery of these natural ion channel modulators via targeted hydrogel-based systems is important due to their potential to enhance therapeutic efficacy while simultaneously reducing systemic toxicity.

In another study, chlorotoxin-modified doxorubicin-loaded 100 nm liposomes increased targeted accumulation and suppressed tumor growth in U87MG glioma cells [219]. In addition, chlorotoxin (CTX)-functionalized temozolomide (TMZ)-loaded chitosan nanoparticles increased the cytotoxic activity of TMZ by increasing its targeted transport in glioblastoma cells [220].

While some current studies focus solely on drug delivery properties, only a limited number of studies have evaluated the functional effects of hydrogel-based carrier systems on cancer cells. This is significant because it demonstrates that the delivery of ion channel modulators via hydrogel systems is feasible. The limited research in this area indicates that the potential of hydrogel-based drug delivery systems for ion channel-targeted cancer therapy has not yet been fully realized.

The therapeutic performance of hydrogel-based ion channel modulator delivery systems is influenced not only by the pharmacological activity of the loaded agents but also by the delivery characteristics of the hydrogel carrier. Controlled and sustained drug release, improved local drug retention, and reduced systemic exposure have been associated with enhanced therapeutic efficacy and reduced toxicity in several of the reported studies. This may be attributed to the ability of hydrogel systems to maintain therapeutic drug concentrations at the target site for prolonged periods while reducing off-target exposure. Prolonged local exposure may also support more sustained modulation of ion channel-mediated processes involved in cancer cell proliferation, migration, invasion, and apoptosis. These findings suggest that hydrogel design may play an important role in determining therapeutic outcomes.

Although the number of available studies remains limited, current evidence indicates that hydrogel-based delivery systems can promote sustained drug release, enhance antitumor efficacy, and, in certain cases, reduce systemic adverse effects. These findings support the further development of hydrogel-based approaches for ion channel-targeted cancer therapy.

**Table 3:** Studies on the delivery and effects of ion channel modulators using hydrogel-based carrier systems.

Hydrogel	Modulator	Target Channel	Cancer Model	Study Type	Effect	Ref.
Chitosan/Pluronic-based injectable hydrogel ((intratumoral/peritumoral; 72 h sustained release)	NS1643	Kv11.1 activator	Triple-negative breast cancer	in vitro/in vivo	Tumor growth ↓	[195]
Xanthan/chitosan hydrogel(topical delivery; sustained re-lease)	Lidocaine	Na^+^ channel blocker	Melanoma	in vitro/in vivo	Tumor growth ↓, Cytotoxicity ↑	[197]
cRGDfk modified nanogel(intravenous; pH-responsive, 72 h release)	Lidocaine	Na^+^ channel blocker	Breast cancer	in vitro/in vivo	Tumor growth ↓, Metastasis ↓, Systemic toxicity ↓	[198]
Pluronic F127 hydrogels (intratumoral; sustained release ~24 h)	Ropivacaine	Na^+^ channel blocker	Breast cancer	in vitro/in vivo	Cisplatin efficacy ↑, Antitumor effect ↑	[199]
Acrylamide and 2-carboxyethyl acrylate (CEA-AAm) NP (sustained release 24 h)	Verapamil	Ca^2+^ channel blocker	Ovarian cancer	in vitro	Intracellular drug accumulation ↑, MDR reversal ↑	[200]
Stimulus-responsive short peptide-assembled (PD/VER) nanogels (sustained release 96 h)	Verapamil	Ca^2+^ channel blocker	Ovarian cancer	in vitro	Intracellular drug accumulation ↑, MDR reversal ↑	[201]
HEMA-Pectin nanogels (dof: sustained release, 24 h; azi: burst release, 2–3 h)	Dofetilide, Azimilide,	K^+^ channel blocker	Lung cancer	in vitro	Dofetilide: (dof): Cytotoxicity ↑ + Paclitaxel ↑ Azimilide: (azi) Cytotoxicity ↑ + Paclitaxel ↔	[162]
(PVA-*co*-PAA)/NaCl IPN hydrogel (controlled release, 24 h)	Diltiazem	Ca^2+^ channel blocker	-	-	Not evaluated in cancer models	[202]
Poly (vinyl alcohol) hydrogel (sustained release, 12 h)	Diltiazem	Ca^2+^ channel blocker	-	-	Not evaluated in cancer models	[203]
Pectin-*co*-poly (acrylic acid) hydrogels (pH-responsive, controlled release)	Nifedipine	Ca^2+^ channel blocker	-	-	Not evaluated in cancer models	[204]
N-succinyl chitosan(Suc-Chi)/alginate hydrogel (controlled release, 15.5 h)	Nifedipine	Ca^2+^ channel blocker	-	-	Not evaluated in cancer models	[205]
Pectin/modified xanthan gum IPN hydrogel (sustained release, ~8 h)	Diltiazem	Ca^2+^ channel blocker	-	-	Not evaluated in cancer models	[206]
Polycaprolactone/acrylic acid (PCL/AA) hydrogels (pH sensitive; controlled release, 24 h)	Verapamil	Ca^2+^ channel blocker	-	-	Not evaluated in cancer models	[207]
Copolymeric tetrahydroxyborate (COP–THB) hydrogel (subcutaneous injection, sustained release, 56 h)	Lidocaine	Ca^2+^ channel blocker	Mice tumor model	in vitro/in vivo	Tumor growth ↓	[208]
Poly(HEMA) hydrogel (sustained release, 72 h)	Nifedipine	Na^2+^ channel blocker	Breast and pancreatic cancer	in vitro	Cytotoxicity ↑	[210]
PEGylated lipid nanoparticles (intravenous; sustained release)	Bupivacaine	Na^+^ channel blocker	Triple-negative breast cancer	in vitro/in vivo	Tumor growth ↓ Metastasis ↓	[212]
Alginate-based hybrid hydrogel (MSL@LID@SOG) (intrapleural; sustained release, 15 days)	Lidocaine	Na^+^ channel blocker	Orthotopic non-small cell lung cancer mouse model	in vitro/in vivo	NK cell activation ↑, Tumor growth ↓, Malignant pleural effusion (MPE) ↓	[214]
Chitosan-genipin/poly(ε-caprolactone) (CS-GP/PC) hydrogel (controlled release, 36 h)	Bupivacaine	Na^+^ channel blocker	-	*-*	Not evaluated in cancer models	[215]
Natural compounds						
Pectin-dihydrazide/oxidized sodium alginate (Pec-DH/OSA) hydrogel (subcutaneous; sustained release, ~72 h)	MTS	TMEM16A	Lung cancer	in vitro/in vivo	Cell growth ↓, Migration ↓, Invasion ↓, Apoptosis ↑	[216]
Oxidized pectin/carboxymethyl cellulose- acylhydrazide (pec-CHO/CMC-AH) self-healing hydrogel (subcutaneous; sustained release)	Limonin	TMEM16A	Lung adenocarcinoma	in vitro/in vivo	Antitumor effect ↑, Proliferation ↓	[217]
Pectin hyrazi-de/oxidized carboxymethyl cellulose (pec-H/DCMC) hydrogel (injectable; sustained release)	Silibinin	TMEMa16A	Lung adenocarcinoma	in vitro/in vivo	Tumor growth ↓, Antitumor effect ↑	[218]

Arrows indicate the following: ↑ increased effect, ↓ decreased effect, ↔ no difference.

## 7. Functional Advantages and Current Limitations of Hydrogel Systems

Macrogel, microgel, and nanogel systems are versatile hydrogel platforms suitable for various biomedical applications based on their dimensional properties. Macrogels are advantageous for tissue engineering and wound dressings because of their high structural integrity and strength. Microgel and nanogel systems, on the other hand, provide controlled drug release and targeted delivery thanks to their improved surface area and diffusion [221].

One of the important advantages of macrogels is their high structural integrity. Furthermore, their ability to create an aqueous microenvironment that can protect cells, ease of modification with biological ligands, and design as systems that can gel at body temperature after injection offer significant advantages in terms of tissue engineering. Recent studies have shown that nanoparticles, bioceramics, and biological agents can be incorporated into injectable and composite hydrogel systems [222]. Injectable macrogel systems can be injected in liquid form and then gel in situ under physiological conditions to fill irregular tissue spaces. This enables the local transport of growth factors and therapeutic agents to the target area. In addition, composite hydrogels enhanced with nanoparticles or bioceramic additives offer significant advantages in terms of increased mechanical strength and controlled drug release [223]. Simultaneously, the dense network structure of macrogels can limit controlled drug release, making it difficult to load drugs or cells homogeneously. Furthermore, mechanical fragility, sterilization difficulties, and structural degradation during long-term use have been observed in some systems. Therefore, recent research has focused on microgels and nanogels.

Microgels are micrometer-sized hydrogel materials that offer a higher surface area and more precise release properties than macrogels. With their high surface area, they facilitate nutrient and metabolite transfer; with their stimuli-sensitive structures, they provide controlled drug release depending on the pH, temperature, or the presence of enzymes [224]. pH-sensitive microgels, developed by exploiting the acidic nature of the tumor microenvironment, enable the controlled release of anticancer drugs in the target area. In addition, hyaluronic acid-based systems can provide selective cellular transport by targeting receptors, such as CD44 [225].

Recent advances in microgel technology have led to the development of stimulus-responsive and multifunctional delivery systems for cancer therapy. These platforms can respond to tumor-specific stimuli such as pH, enzymatic activity, or redox conditions, enabling controlled and site-specific drug release. Furthermore, multifunctional hydrogel systems capable of co-delivering therapeutic agents, genes, or immune modulators have shown enhanced antitumor efficacy compared with conventional delivery approaches. Such systems represent a promising strategy for improving treatment outcomes while reducing systemic toxicity [226,227]. However, the microgel production process can be complex. Homogeneous control of the particle size is difficult. For these reasons, although low crosslinking increases biocompatibility, it reduces mechanical stability and may pose an obstacle in clinical applications due to limited long-term in vivo safety data.

Nanogels are nanometer-sized hydrogel systems that are among the most advanced platforms for targeted drug delivery and smart therapeutic applications. Owing to their nano dimensions, they can be easily taken up by cells, passively or actively targeted to tumors, and can increase bioavailability, providing significant advantages [228]. Owing to their network structure, nanogels enable the delivery of hydrophilic and hydrophobic drugs. Nanogels have a wide range of applications in fields such as chemotherapy, gene delivery, vaccine development, and neurodegenerative disease treatment [229,230]. They can help overcome biological barriers, particularly in intranasal, ocular, and pulmonary applications. In addition, the ability to load multiple drugs simultaneously provides a significant advantage for combination therapies. Smart nanogel systems developed in recent years are designed to be sensitive to pH, temperature, redox environment, or enzyme presence and can provide controlled drug release [231]. Redox-sensitive nanogels that respond to biological stimuli, such as glutathione (GSH), which is found at high levels in tumor cells, are site-specific and promising for drug delivery [232].

In addition, nanogel systems developed using 3D printing technologies provide next-generation approaches for personalized treatment, tissue engineering, and biomedical implant applications. However, the production of nanogels is complex and expensive. Their small size can lead to problems, such as aggregation, loss of stability, and rapid biological clearance. Furthermore, the long-term toxicity and clinical safety of nanomaterials are not fully understood. The lack of large-scale production and clinical trials limits the industrial application of nanogels [233].

Overall, macrogels offer high mechanical strength and structural stability, whereas microgels provide advantages in terms of controlled release, cell transport, and injectability. Nanogels are notable for their applications in targeted drug delivery, bioavailability, and stimulus-responsive smart therapy systems. However, each hydrogel system has specific limitations in terms of mechanical strength, fabrication complexity, sterilization, toxicity, and clinical applicability. When considering each hydrogel class, degradation byproducts may raise safety concerns related to potential immune responses and long-term biocompatibility. These aspects should be carefully evaluated before clinical translation. Consequently, the selection of a suitable hydrogel system should be optimized according to the targeted biomedical application, drug type, biological environment, and desired release behavior.

## 8. Future Perspective

While the importance of ion channels in cancer biology is increasingly understood, the translation of therapeutic approaches targeting these channels to clinical practice still faces limitations. Distinguishing between healthy and malignant cells based on their ion channel expression profiles could enable the development of a selective and multifaceted treatment approach [137]. However, because it is difficult to implement this selectivity consistently in systemic applications, hydrogels that provide targeted and controlled drug release have come to the forefront. Hydrogel-based platforms contribute to optimizing regional therapeutic efficacy and reducing off-target biological effects by enabling the controlled release of ion-channel modulators. In this regard, hydrogel delivery systems can enable the remodulation of the tumor microenvironment by allowing the combined use of ion channel blockers and modulators that support the activation of immune cells in cancer cells [234]. The functional effects of ion channels in the tumor microenvironment and tumor cells necessitate the evaluation of these channels as multi-system components. The tumor microenvironment, with its multi-component organization, is particularly important for therapeutic targeting in cancer studies, especially because pH and hypoxic conditions reshape ion channels [235]. Smart hydrogel systems, which are sensitive to changes such as tumor microenvironment-specific enzymatic activity and pH, offer significant potential for enhancing the target-specific selectivity of ion channel modulators and improving treatment efficacy. Such smart systems have the potential to enhance treatment efficacy by controlling drug release at the desired time and for the desired duration [45].

Nanoparticle-hydrogel hybrid systems also offer remarkable advantages, such as increased drug stability and the ability to co-deliver multiple agents. Thus, they offer new opportunities in terms of achieving high therapeutic efficacy and reducing off-target effects in the elimination of tumor tissue [137]. In addition, simultaneous or sequential combination therapy with chemotherapeutic agents and ion channel modulators can overcome drug resistance in cancer cells and increase antitumor efficacy. In addition, the controllable size of the targeted hydrogel-based systems can reduce unwanted distribution in healthy tissues and increase their accumulation in the tumor region with increased permeability and retention (EPR) effect [200].

In the future, hydrogel-based drug delivery systems should be optimized for biocompatibility, size, interaction with biological barriers, controlled release, and clinical needs. This will improve the effectiveness and safety of these approaches. A significant portion of future research is expected to focus on smart hydrogel systems with stimulus-response properties. Nanogel platforms sensitive to biological signals such as pH, temperature, glutathione concentration, or tumor-specific enzyme activity offer a major advantage in providing time- and target tissue-dependent controlled release of therapeutic agents [236,237]. This enables much more precise and personalized treatment options compared with traditional drug applications. This approach offers significant potential, particularly for ion channel blockers, chemotherapeutics, and biological agents with a high risk of systemic toxicity. Simultaneously, the development of controlled release mechanisms can lead to safer and more effective options for the treatment of chronic pain, cardiac arrhythmias, cancer, and neurological diseases. These advancements could be significant steps towards providing individuals with a higher quality of life. Furthermore, smart nanogel systems offer synergistic treatment by combining chemotherapy, immunotherapy, phototherapy, and gene therapy [238,239]. These systems view the heterogeneous structure of the tumor microenvironment not only as a biological barrier but also as a trigger for promoting controlled release. In particular, the development of redox-sensitive or enzyme-sensitive platforms has become a significant research topic in individual cancer treatment. Moreover, because surface-functionalized nanogels show promise in central nervous system diseases by targeting transcytosis via receptors, nanogel strategies aimed at overcoming the blood–brain barrier are expected to gain even more importance in the future. However, the fact that in vitro results are not always consistent with in vivo pharmacokinetics is a challenge encountered in clinical applications. Future research should focus on the biodistribution, biodegradation, and long-term safety parameters of these nanomaterials.

Although hydrogel-based drug delivery systems have shown considerable promise in preclinical studies, several challenges remain before their widespread clinical implementation can be achieved. Future research should focus on improving the reproducibility, scalability, long-term biosafety, and clinical translation of these platforms. In addition, a better understanding of the interactions between ion channel modulators, hydrogel carriers, and the tumor microenvironment may facilitate the development of more effective and personalized therapeutic strategies. The integration of targeted delivery, controlled release, and combination therapy within a single platform is expected to be a major direction for next-generation hydrogel-based cancer treatments. Furthermore, the use of clinically relevant vivo models and comprehensive long-term safety studies will be essential for accelerating the translation of these systems from laboratory research to clinical applications.

Overall, hydrogel-based drug delivery systems are expected to evolve into smarter, more adaptable, and multifunctional platforms. The development of systems capable of overcoming biological barriers, responding to disease-specific stimuli, and adapting to individual patient needs is likely to shape the future of this field. Hydrogel-based strategies for the delivery of ion channel blockers and modulators may provide new opportunities for precision medicine and targeted cancer therapy. The continued integration of advanced biomaterials, nanotechnology, and controlled release approaches is expected to further expand the therapeutic potential of hydrogel systems in the coming years.

## 9. Conclusions

Preclinical studies on ion channel modulators (activators or inhibitors) have shown promising results. As previously described, these represent potential therapeutic strategies that enhance the effectiveness of cancer treatment. Target-specific delivery of these ion channel modulators via hydrogel-based carriers can increase the sensitivity of cancer cells to conventional chemotherapeutic agents and offer complementary treatment strategies. They also provide innovative approaches to make the immune system’s response to cancer cells more effective. Ion channel modulators delivered via hydrogel-based systems can increase antitumor efficacy, overcome drug resistance, reorganize the complex tumor microenvironment, and improve immunotherapeutic responses. However, further research is needed to support these findings and their clinical translation.

## Figures and Tables

**Table 2:** Comparison of the advantages and clinical translation challenges associated with macrogel, microgel, and nanogel systems.

Hydrogel Type	Advantages	Clinical Translation Challenges
Macrogels	High drug loading capacity, structural stability, suitability for localized therapy	Requirement for implantation or surgical placement, limited tissue penetration
Microgels	Injectable administration, controlled drug release, good biocompatibility	Batch-to-batch reproducibility, sterilization, and scalability challenges
Nanogels	Enhanced tissue penetration, improved cellular uptake, potential for targeted delivery	Complex manufacturing processes, long-term safety concerns, regulatory approval requirements

## Data Availability

Not applicable.
